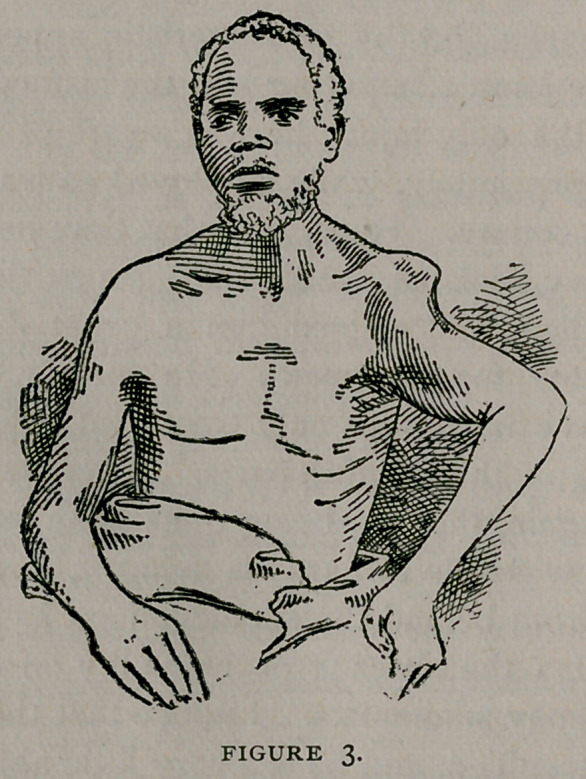# Surgical Cases

**Published:** 1888-08

**Authors:** W. Z. Holliday

**Affiliations:** Harlem, Ga.


THE ATLANTA MEDICAL AND SURGICAL JOURNAL.

VOL. V. AUGUST, 1888. NO. 6.

### Original Communications.

### SURGICAL CASES.

1. FRACTURE OF THE SKULL, WITH CONSIDERABLE LOSS OF BRAIN SUBSTANCE—RECOVERY WITH NO UNFAVORABLE SYMPTOMS. 2. FRACTURE AND DISLOCATION OF HUMERUS, WITH FALSE JOINT—RESULT OF NATURE'S SURGERY*.

BY W. Z. HOLLIDAY, M. D., HARLEM, GA.

CASE I.—April 11th, 1887. I was called this afternoon to see L. H., negro, æt. about 40. He has just been engaged in a fight, during which he was felled to the ground by a blow on his head with a stick. I find him in a deep stupor, with slow, labored pulse and irregular respiration. The blow from which he suffers was received in the left temporo-parietal region, just above and slightly in front of the ear. At this point there is an evident depression, and, on close inspection, I can see particles of brain

* Read by title at 39th Session Medical Association of Georgia.

substance around the wound. My attention is next given to his mouth, which I notice is bloody, and covered with sand. Forcing his jaws apart, I find that his tongue is almost completely severed by an ugly gash across the centre of its upper surface. I learn from those who witnessed the tragedy that he fell heavily to the ground when he was struck, and his face contained several scratches, evidently produced by the fall. The skin on his chin showed the impressions of coarse gravel, and it is evident that a large part of his weight, and the impetus of the fall, were received at this point, and the tongue being caught between the teeth, was injured as already described. I now cleanse the parts thoroughly, and having all foreign material removed, the tongue is pulled forward, the edges of wound are brought together and retained in position by the application of five cat-gut sutures. The head, having also been cleansed and the wound examined, is now covered with light bandage, and I leave him to wait for a reaction before using any further operative measures.

April 12th. This a.m. found him doing very well. He slept last night, and has taken a glass of milk this morning. There is some stupor, though he can be easily aroused. There is slight dilation of pupils, though it is about even in each. The bandage is now removed from his head, and on exploring the scalp wound with my little finger, I find a considerable button of bone deeply depressed. The patient is next etherized, and the scalp wound is enlarged in order to make a more thorough examination, and institute whatever operative measures may be necessary. Coming down upon the bone, the fracture is plainly outlined, and the depression is very evident, as is also the fact that meninges are ruptured and some of the cortical portion of the brain has been lost. With an elevator I managed, after several unsuccessful efforts, to raise the depressed button of bone, which I find measures one and one-half inches in its long diameter. The difficulty in removing this was due to the fact that fracture was larger on inner table than on the outer. A considerable number of smaller fragments of bone are now easily detached from the margin of opening. When these are removed, a considerable quantity of brain substance, which had been detached by force of blow, now oozes away. The amount was estimated by myself and assistant to be fully two tablespoonsful. The hemorrhage, which begun with removal of bone, was controlled by application of cat-gut ligatures to the bleeding vessels. I next removed all the sharp spiculæ of bone from the margin of opening, which might later be a source of irritation to the brain. This being accomplished, the wound is thoroughly irrigated with a 1-2000 sol. bichloride mercury, and is lightly covered with a compress of absorbent cotton, in order to be quite certain that all hemorrhage is controlled before closing the scalp wound. The patient having been allowed time to recover from effects of ether, now declares that he feels comfortable, and I leave him. Returning in six hours, the compress is removed, and I find that all hemorrhage had been controlled. I proceed to insert drainage-tube, and closed the wound with several sutures of silver wire. Pulse is now 70, temp. 101, respiration good, and I give him 10 grs. quinine.

April 13th, 10 a. m. Find him comfortable and rational; able to sit up on bed and talk. Pulse 80, temp. 100 4-5, resp'r 20. Complains of some uneasiness about his mouth, caused by swollen condition of the tongue. Says he is hungry. I order for him to-day 8 grs. quinine, every three hours, and a generous diet of gruel and sweet milk. There is considerable swelling on right side of his face—almost enough to close the eye. His general condition is good.

13th, 8 p. m. Pulse 77, temp. 100 4-5, resp'r 19. Says he has suffered some during the day with pain in his head, and that it still troubles him. In order to relieve him of this, and to secure rest for the night, I give him ¼ gr. morphine subcutaneously. His bowels not having acted since injury, I order a dose of Epsom salts to be given at bed-time, and quinine in 8 gr. doses at six and twelve to-morrow. Milk diet is continued.

April 14th, 4 p. m. Pulse 74, temp. 100, resp'r 18. Complains of soreness about head and on outer side of right hip.

16th. Pulse 70, temp. 99½, resp'r 22. General condition good. Tongue is now healed. He has been nervous and restless, and I give him brom. potass. 20 grs. ter in die. He is now taking a plenty nourishment, consisting of milk and chicken broth.

18th. Pulse 76, temp. 99, resp'r 20. Comfortable.

20th. Pulse 75, temp. 98, resp'r 20. The dressing is now removed from his head, and I find the wound is nicely united. Sutures and tube are removed and the parts are dry and healthy, there being no discharge whatever, I apply plenty iodoform (as had been done with first dressing) and re-apply the dressings.

27th. Dressing is removed again to-day, and parts are dry and clean, not one drop of pus. He is now dismissed, convalescent. During the course of treatment there was no decided systemic disturbance. This, I think, is a little remarkable, when we consider the nature of injury and amount of brain substance lost. The highest temperature was recorded on second day, just after operation, and was 101. This probably went higher than it would have been without the operative interference. At one time there was some loss of power in muscles of expression on opposite side of face, but this was soon restored. In a few weeks he seemed just as well as he ever was in his life. I have seen him several times since, and he has had no trouble resulting from the injury. The opening in cranium is pretty well filled with a cartilaginous mass which protects the brain well. There was never any change in his mental condition after he recovered from the shock. In the management of this case, antiseptic precautions were observed as strictly as possible, carbolic solution being used for all instruments, and the bichloride mercury 1-2000 for irrigation. There was never sufficient rise of temperature to occasion discomfort, and the union, both in tongue and scalp, was complete, and by first intention.

CASE 2.—This case is presented because of its rarity and of the interest that might attach to it as showing how nature acts as a surgeon. The case came under my observation five years after injury, and rather accidentally then, for I was called to treat him for a constitutional and not the local disorder. John L., aged 65, had recently come from an adjoining county. Called to see him at his home, and found him suffering with muscular rheumatism. The trouble, while more or less general, was worse in left arm and shoulder, and it is only with great pain and difficulty that he can move these parts at all. Having been stripped of his clothing, I found existing such a condition of things as is shown in fig. No. 1. Here we can see on front side

FIGURE 1.

the bony landmarks and the characteristic appearance of a dislocated shoulder joint. Inquiring into the history of this trouble, I am told that the only injury he was aware of having received was five years previously, while employed as wagon driver on a farm in Lincoln county. He says at that time he was hurt while handling a heavy stick of wood. He states that his arm was very much swollen, and pained him a great deal, and that he went to a doctor for treatment. He further stated that the medical man gave him, as his only treatment, a bottle of liniment with which to rub the painful parts. After several weeks he went to work again, though, being quite lame on those parts for a long time, he was able to earn his living. Now looking at fig. 2, you can see from behind the shoulder joint as I found it. You observe here also the absence of rotundity on outside and the prominence of bony landmarks. I notice that the deltoid muscle is very much atrophied, and he has very little ability to bring the

FIGURE 2.

arm up in vertical direction. He says this is as it has been quite a long time. On searching for the head of humerus, I find it separated from the bone about one inch below its neck, and hid away in the subscapular fossa. I find also on manipulating the

FIGURE 3.

arm that there is a false joint established some three inches below the shoulder joint proper. See fig. 3. I treated his rheumatism with salicylate soda, and he did well, and was out in a few days. After he was able to come to my office, I applied the faradic current to his shoulder, and he improved rapidly, and soon had much better use of the arm. In a little while he resumed his occupation. I have seen him a great many times since, and he goes about his business and does any light work without special inconvenience.

The last time I saw him he was in a cotton field, and was hoeing away at a lively rate. In the foregoing history, I am unable to vouch for the truthfulness of statements made to me. The interest that attaches to this case would be the *result* rather than the *cause* or method of obtaining. It goes to show how cleverly, yet how awkwardly, nature, when unaided, acts as a surgeon.

## Figures and Tables

**FIGURE 1. f1:**
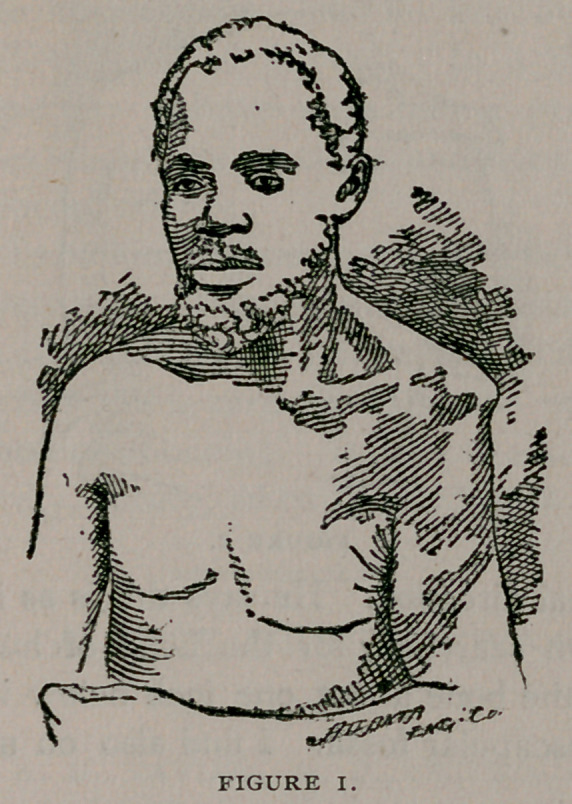


**FIGURE 2. f2:**
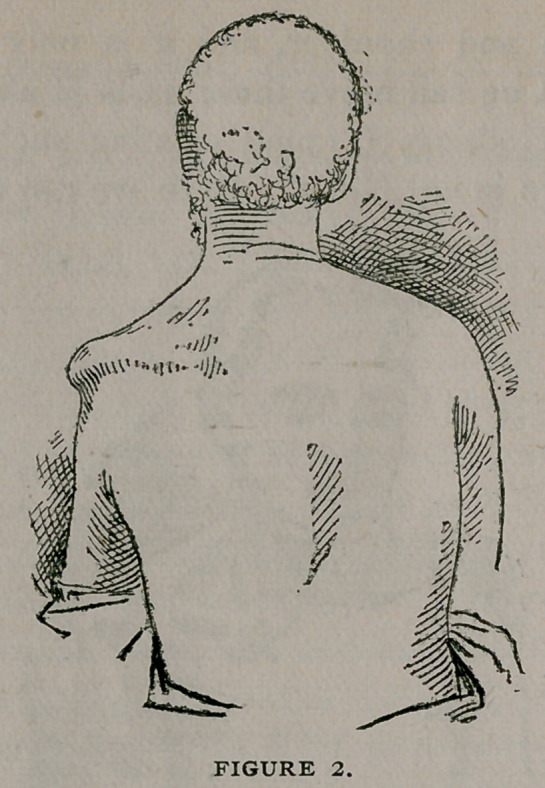


**FIGURE 3. f3:**